# Resolution of Ortner’s Syndrome Following Successful Mitral Transcatheter Edge-to-Edge Repair: The First Case Report

**DOI:** 10.7759/cureus.102949

**Published:** 2026-02-04

**Authors:** Ukachukwu I Okoro, Rajesh Varma, Benoy N Shah, Eunice N Onwordi

**Affiliations:** 1 Cardiology, University Hospital Southampton NHS Foundation Trust, Southampton, GBR

**Keywords:** cardiovocal syndrome, case report, mitral regurgitation, ortner’s syndrome, transcatheter edge-to-edge repair

## Abstract

Ortner’s syndrome (OS), or cardiovocal syndrome, is an uncommon cause of hoarseness caused by recurrent laryngeal nerve palsy due to compression from underlying cardiovascular disease. It is classically associated with left atrial enlargement due to rheumatic mitral stenosis and, less frequently, severe mitral regurgitation. We present the case of an 82-year-old man with progressive dyspnea and hoarseness who was found to have severe mitral regurgitation and marked left atrial dilation. Following successful transcatheter edge-to-edge mitral repair, he experienced significant improvement in cardiac symptoms and vocal quality. To the best of our knowledge, this is the first reported case of OS resolving after transcatheter mitral edge-to-edge repair.

## Introduction

Ortner’s syndrome (OS), also known as cardiovocal syndrome, is a rare condition in which left recurrent laryngeal nerve (RLN) palsy occurs secondary to underlying cardiovascular pathology. It was first described in 1897 by the Austrian physician Norbert Ortner, who identified an association between mitral stenosis with left atrial enlargement and hoarseness resulting from compression of the left RLN [1].

Since Ortner’s initial description, numerous other causes of OS have been reported in the literature, including aortic aneurysms, pulmonary hypertension, and congenital heart disease [2]. Left atrial volume overload resulting from severe mitral regurgitation can likewise lead to left atrial enlargement, producing similar sequelae [3]. Resolution of hoarseness in OS is uncommon and depends on the duration of RLN injury and the reversibility of the underlying cardiovascular cause [4,5].

This case highlights the resolution of hoarseness in OS following mitral transcatheter edge-to-edge repair (mTEER), which, to the best of our knowledge, represents the first reported case in the literature.

## Case presentation

**Figure 1:**
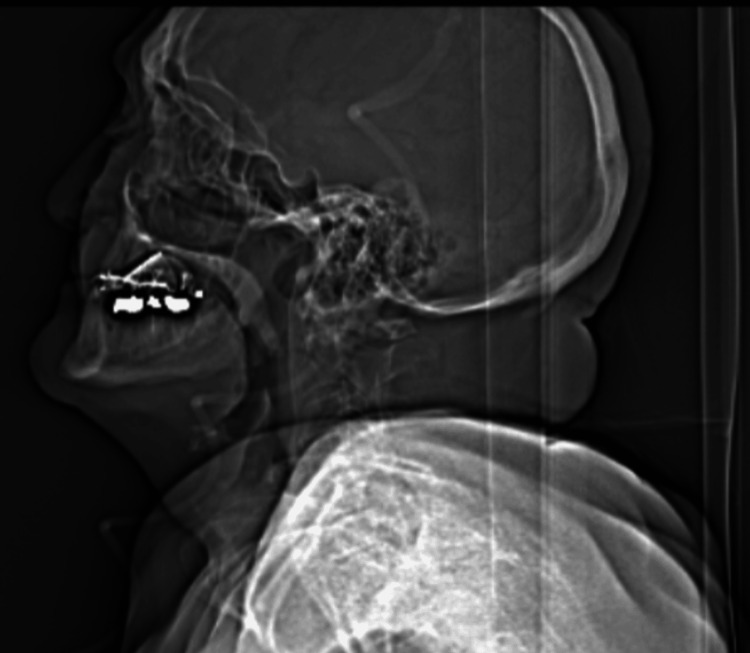
CT scan of the neck demonstrating no discernible mass or focal abnormality within the larynx or elsewhere within the upper aerodigestive tract

An 82-year-old man was referred to the cardiology team with a history of worsening shortness of breath on exertion. His medical history included a recent hospital presentation with pulmonary congestion and a two-month history of hoarseness for which he had been referred to the ENT team. The vocal hoarseness developed around the same time as the onset of dyspnea. He was found to have left vocal cord palsy and underwent extensive evaluation for hoarseness, including a CT scan of the neck, which showed no space-occupying lesion (Figure 1).

**Figure 2:**
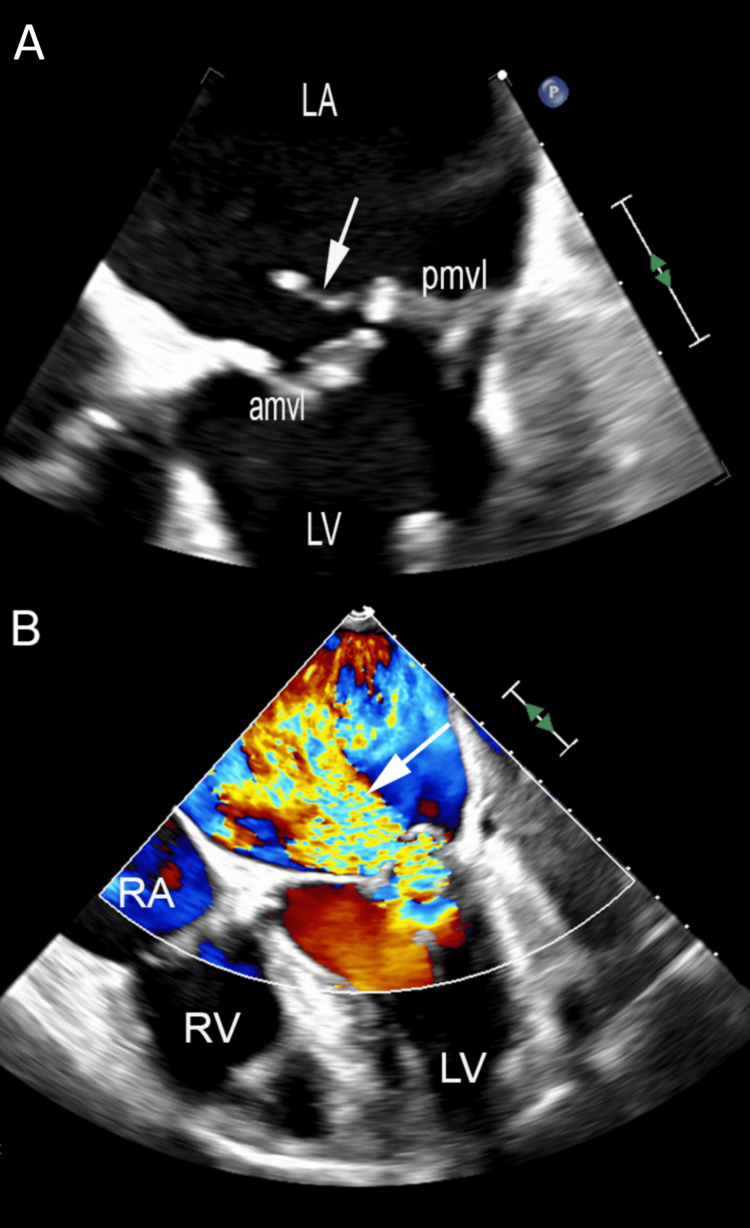
Transesophageal echocardiogram (A) Four-chamber view showing a dilated LA cavity. A ruptured mitral valve chord (white arrow) with a flail P2 scallop is seen. (B) Color Doppler assessment demonstrating subsequent very severe anteriorly directed mitral regurgitation. AMVL, anterior mitral valve leaflet; LA, left atrium; LV, left ventricle; PMVL, posterior mitral valve leaflet; RA, right atrium; RV, right ventricle

**Figure 3:**
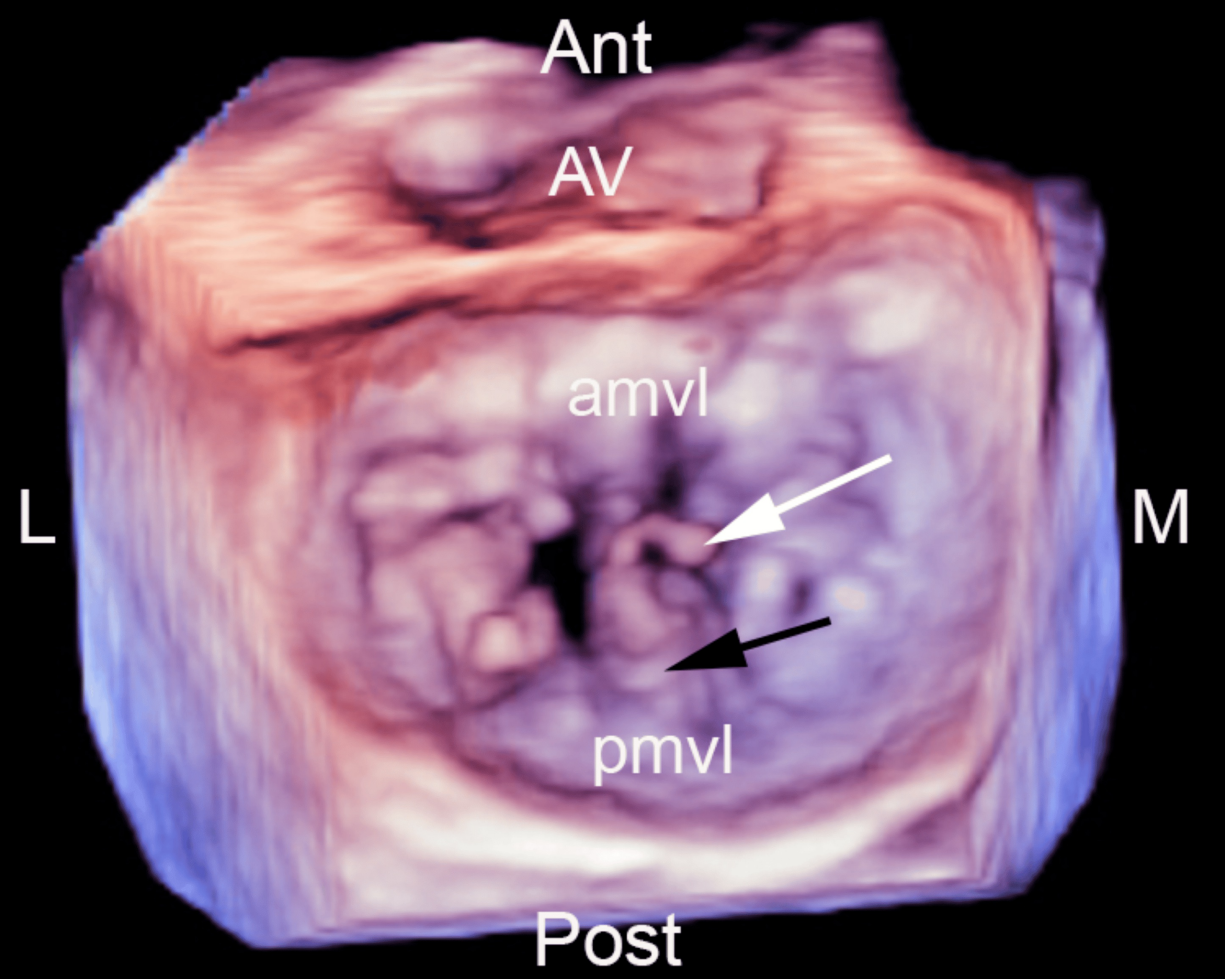
Three-dimensional mitral valve reconstruction demonstrating the flail P2 scallop and ruptured chordal mechanism AMVL, anterior mitral valve leaflet; Ant, anterior; AV, aortic valve; L, lateral; M, medial; PMVL, posterior mitral valve leaflet; Post, posterior

A transthoracic echocardiogram demonstrated a flail posterior mitral valve leaflet with ruptured chordae, resulting in very severe eccentric mitral regurgitation (Figure 2, Figure 3). The left atrium (LA) and left ventricle were dilated. Left ventricular systolic function was dynamic in the setting of very severe mitral regurgitation, and pulmonary artery pressure was elevated. No other significant abnormalities were identified. It was felt that acute chordal rupture of the mitral valve was responsible for the recent onset of dyspnea. Following extensive review by the ENT and cardiology teams, his hoarseness was attributed to severe mitral regurgitation, causing a rapid rise in left atrial pressure and progressive left atrial dilation.

He was treated with bumetanide 1 mg once daily but had minimal symptomatic response, and his hoarseness remained unchanged. Following discussion with the heart team, he was accepted for mTEER.

**Figure 4:**
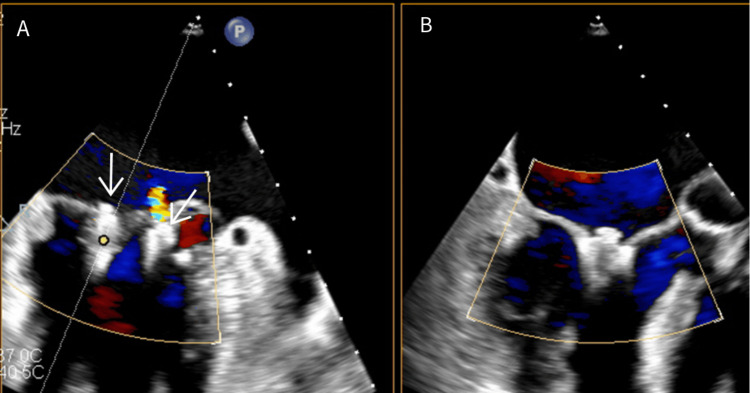
Transesophageal echocardiogram (A) Two mitral valve edge-to-edge devices inserted centrally between A2/P2 and laterally (white arrows). (B) Demonstration of only mild residual mitral regurgitation.

**Figure 5:**
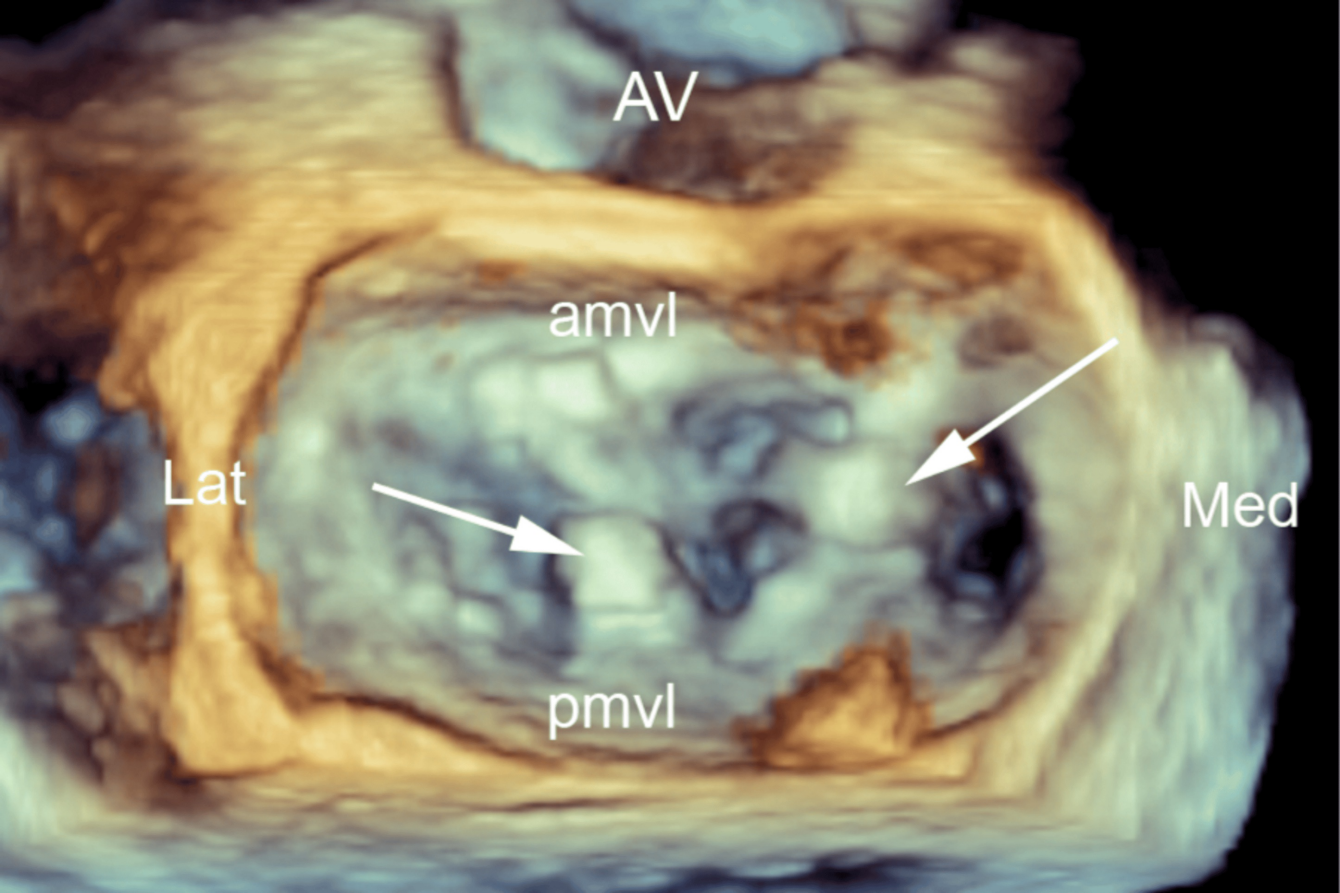
Three-dimensional mitral valve reconstruction demonstrating mTEER device deployment and resolution of the flail P2 scallop AMVL, anterior mitral valve leaflet; AV, aortic valve; Lat, lateral; M, medial; mTEER, mitral transcatheter edge-to-edge repair; PMVL, posterior mitral valve leaflet

He underwent a successful procedure with one PASCAL device (Edwards Lifesciences, Irvine, CA, USA) deployed at A2/P2 and an additional lateral device, reducing mitral regurgitation from very severe to mild (Figure 4, Figure 5). The mean mitral gradient was 5 mmHg at the end of the procedure. Left atrial pressure decreased by 3 mmHg. Although this reduction was modest, it likely reflected complex left atrial hemodynamics influenced by reduced left ventricular compliance and severe atrial enlargement. Normalization of pulmonary vein flow during the procedure was reassuring.

At follow-up in the heart valve clinic, the patient reported improvement in cardiac symptoms, with no significant dyspnea during day-to-day activities. Notably, he also reported a rapid improvement in vocal quality within weeks of the transcatheter edge-to-edge repair (TEER) procedure. Although some residual hoarseness persisted, repeat laryngoscopy demonstrated ongoing vocal cord palsy, and he was subsequently referred for speech therapy.

## Discussion

OS classically describes vocal cord paralysis secondary to left RLN compression due to underlying cardiovascular pathology. The left RLN’s course through the aortopulmonary window makes it more susceptible than the right RLN to compression by surrounding structures [2,6]. Mitral valve disease, particularly when associated with left atrial enlargement, has traditionally been described as a primary cause [1,2]. Most reported cases have involved mitral stenosis, whereas this case demonstrates left atrial enlargement from severe mitral regurgitation, which is less commonly reported.

Recovery of vocal cord function may depend on the duration and severity of RLN compression [4,5]; however, this is variable. For example, Acharya et al. described a patient with a giant LA who regained her voice within one week of double valve replacement surgery [7], whereas Stoob et al. reported a patient whose hoarseness resolved one year after endovascular repair of a thoracic aortic aneurysm [8]. In both cases, symptoms had been present for several months. Most reports of OS involve underlying cardiovascular causes requiring open surgical procedures or endovascular repair. To the best of our knowledge, our case demonstrates, for the first time, resolution after percutaneous mTEER.

This highlights that percutaneous therapies such as TEER are not only effective for symptomatic relief in patients with severe mitral regurgitation but can also potentially address rare extracardiac complications of mitral valve disease, such as hoarseness. Although definitive management of OS centers on correction of the underlying cardiovascular pathology, speech therapy may play an important supportive role in patients with persistent dysphonia. Previous case reports have described meaningful improvement in vocal quality following structured speech therapy after treatment of the causative cardiovascular lesion [9,10].

In our patient, despite rapid improvement in vocal quality following TEER, residual hoarseness and persistent vocal cord palsy were observed. Referral for speech therapy was therefore appropriate, demonstrating the value of a multidisciplinary approach to management.

This case contributes to existing evidence of reversal of hoarseness in OS following intervention for the underlying cardiovascular disease, further emphasizing the importance of timely correction of the causative pathology before irreversible nerve damage occurs.

## Conclusions

OS remains a rare manifestation of cardiac disease, and resolution following treatment of the underlying pathology is uncommon. The rapid improvement in hoarseness following mitral repair underscores the importance of timely intervention for underlying cardiovascular pathology in these patients. To the best of our knowledge, this case represents the first documented reversal of OS following mTEER, illustrating the benefits of minimally invasive interventions as safe and effective alternatives to open surgery, not only for restoring cardiac function but also for treating rare extracardiac complications.
